# Successful treatment of refractory primary follicular mucinosis with Tofacitinib

**DOI:** 10.1016/j.abd.2026.501351

**Published:** 2026-04-23

**Authors:** Yu-Lian Li, Sheng Fang

**Affiliations:** Department of Dermatology, the First Affiliated Hospital, Chongqing Medical University, Chongqing, China

Dear Editor,

Follicular Mucinosis (FM), a rare cutaneous mucinosis, is characterized by erythematous infiltrated plaques with follicular prominence. There is no consensus on the treatment of FM. Current therapeutic options include mild-to-moderate potency topical corticosteroids, topical and oral antibiotics, retinoids, dapsone, sulfacetamide, imiquimod, pentoxifylline, pimecrolimus, Psoralen Plus Ultraviolet A (PUVA), and nitrogen mustard.^1^ Emerging evidence suggests that tofacitinib may be effective for cutaneous mucinoses, with reported efficacy in both pretibial myxedema and refractory papulonodular mucinosis.^2^, ^3^ Here, we report a patient with refractory FM who was successfully treated with tofacitinib.

A 55-year-old female patient presented to the dermatology clinic with a 4-month history of a right facial plaque. She denied any topical application, drug intake, or photosensitivity. Her systemic and family history was unremarkable. Physical examination demonstrated a poorly demarcated, edematous, erythematous plaque on the right cheek. (Fig. 1A) No other cutaneous involvement was observed. According to clinical and histopathological features, the patient was diagnosed with Primary Follicular Mucinosis (PFM). Initial treatment with hydroxychloroquine (200 mg twice daily) and high-potency topical corticosteroids (clobetasol propionate 0.05% ointment) resulted in no clinical improvement after 8-weeks of therapy. Monotherapy with tofacitinib 5 mg twice daily was started. After 3-months of treatment, significant clinical improvement was observed, manifested by complete resolution of skin lesions (Fig. 1B). No recurrence was observed during the three-month follow-up period following treatment discontinuation. Histopathological examination revealed the accumulation of mucin in pilosebaceous follicles and sebaceous glands, with perifollicular infiltrates of lymphocytes and eosinophils observed (Fig. 2A). Alcian blue staining confirmed mucin deposition within the follicular epithelium (Fig. 2B). Immunohistochemical analysis demonstrated that lymphocytes were positive for Leukocyte Common Antigen (LCA), Ki-67 (20% positivity rate), CD8, CD20, CD7, CD79a, CD4, and CD3, with a balanced CD4^+^/CD8^+^ ratio. (Figs. 2C–D) The result of the T-cell Receptor (TCR) gene rearrangement was negative.

**Figure 1:**
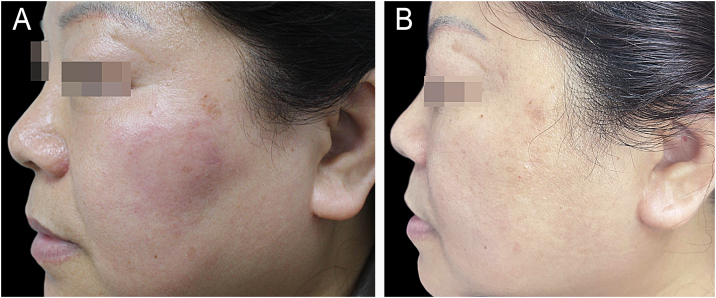
(A) A poorly demarcated edematous erythematous plaque was observed on the right cheek. (B) Complete clinical resolution was achieved after 3-months of tofacitinib monotherapy.

**Figure 2:**
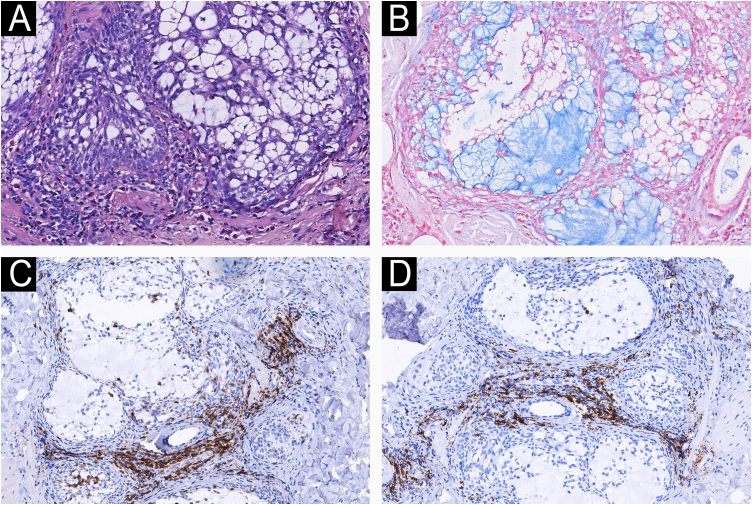
(A) Massive mucin deposition in the follicular epithelium (Hematoxylin & eosin, ×40). (B) Deposition of mucin confirmed by Alcian blue staining (×200). Immunohistochemical analysis demonstrated that lymphocytes were positive for CD4 (C) and CD8 (D) with a balanced CD4^+^/CD8^+^ ratio (×100).

Follicular mucinosis is characterized by the extensive deposition of mucin at the external root sheath and sebaceous glands. FM can be categorized as Primary Follicular Mucinosis (PFM) and Secondary Follicular Mucinosis (SFM). The most common clinical symptom of PFM is a solitary lesion in the head or neck region in young patients. As for the histopathological findings, the presence of extensive mucin deposition in cystic spaces, minimal perivascular and periadnexal polyclonal infiltration of non-atypical lymphocytes, the absence of epidermotropism, and an equivalent CD4^+^/CD8^+^ cell rate pointed towards a diagnosis of PFM. Secondary Follicular Mucinosis (SFM) may be associated with certain medications, benign conditions, and malignancies (most commonly lymphomas). Lymphoma-associated FM typically presents with multiple extracranial lesions in elderly patients. Histopathological examination reveals a dense band-like infiltrate with atypical lymphocytes, epidermotropism, a prominent CD4^+^ immunophenotype, and monoclonal gene rearrangement of the infiltrate.^1^

The pathogenesis of Follicular Mucinosis (FM) remains incompletely understood. Current evidence suggests that follicular keratinocytes may be the source of mucin deposition, a process activated by T-cell-mediated immune mechanisms and cytokines. T-lymphocytes secrete IL-4, IL-2, and IL-26, inducing phosphorylation of Signal Transducer and Activator of Transcription (STAT), thereby affecting keratinocyte function.^4^ Tofacitinib, as a pan-JAK1/JAK3 inhibitor, can interrupt pathological signaling by blocking STAT phosphorylation.^5^ Studies have also demonstrated a significant correlation between the quantity of mucin within hair follicles and the severity of perifollicular inflammatory infiltration. Mediators released by T-lymphocytes, including IFN-γ, IL-5, TGF-β, and IL-4, trigger and amplify inflammatory cascades.^4^ The JAK-STAT pathway plays a vital role in immune regulation, cell differentiation, apoptosis, and proliferation.^5^ By inhibiting JAK1/JAK3, tofacitinib effectively reduces the production of proinflammatory cytokines in T-lymphocytes. Tofacitinib, in our case, was selected primarily due to its comparatively lower cost relative to other highly selective JAK inhibitors. Furthermore, published literature has indicated its promising efficacy in the treatment of mucinous dermatosis.^2^, ^3^

There are currently no established treatment guidelines for follicular mucinosis, and management remains individualized. Available therapeutic options have demonstrated limited efficacy in clinical practice. This pioneering case demonstrates the first successful therapeutic application of JAK inhibitors in follicular mucinosis. While these findings are promising, the inherent limitations of this single-case study underscore the need for validation through large-scale randomized controlled trials. Additionally, further mechanistic investigations are essential to elucidate the precise therapeutic pathways of tofacitinib in FM.

## ORCID ID

Yu-Lian Li: 0009-0004-4608-6548

## Research data availability

Does not apply.

## Financial support

None declared.

## Authors' contributions

Yu-Lian Li: Data curation; Methodology; Formal analysis; Visualization; Validation; Writing-original draft.

Sheng Fang: Conceptualization; Methodology; Resources; Supervision; Writing-review & editing.

## Conflicts of interest

None declared.
